# Impact of CD4 and CD8 dynamics and viral rebounds on loss of virological control in HIV controllers

**DOI:** 10.1371/journal.pone.0173893

**Published:** 2017-04-05

**Authors:** Fanny Chereau, Yoann Madec, Caroline Sabin, Niels Obel, Ezequiel Ruiz-Mateos, Georgios Chrysos, Sarah Fidler, Clara Lehmann, Robert Zangerle, Linda Wittkop, Peter Reiss, Osamah Hamouda, Vicente Estrada Perez, Manuel Leal, Amanda Mocroft, Patricia Garcia De Olalla, Adriana Ammassari, Antonella D’Arminio Monforte, Cristina Mussini, Ferran Segura, Antonella Castagna, Matthias Cavassini, Sophie Grabar, Philippe Morlat, Stéphane De Wit, Olivier Lambotte, Laurence Meyer, The HIV Controllers Project Working Group for the Collaboration of Observational HIV Epidemiological Research Europe (COHERE) in EuroCOORD

**Affiliations:** 1 Université Paris-Saclay, and Université Paris-Sud and Université de Versailles Saint-Quentin-en-Yvelines, and CESP, INSERM U1018, Kremlin-Bicêtre, France; 2 Emerging Diseases Epidemiology Unit, Institut Pasteur, Paris, France; 3 Research Department of Infection and Population Health, UCL, Royal Free Campus, Rowland Hill Street, London, United Kingdom; 4 Department of Infectious Diseases, Rigshospitalet, Copenhagen, Denmark; 5 Laboratory of Immunovirology, Clinic Unit of Infectious Diseases, Microbiology and Preventive Medecine, Institute of Biomedecine of Seville, IBiS, Virgen del Rocío University Hospital/CSIC/University of Seville, Seville, Spain; 6 Department of Medicine, Infectious Disease Unit, Tzaneio General Hospital of Piraeus, Piraeus, Greece; 7 Imperial College, London, United Kingdom; 8 Department of Internal Medicine I, University of Cologne and German Center for Infection Research (DZIF), partner site Bonn-Cologne, Cologne, Germany; 9 Department of Dermatology and Venereology, Medical University Innsbruck, Innsbruck, Austria; 10 University Bordeaux, ISPED, INSERM U1219-Bordeaux Population Health, INSERM, ISPED, Centre INSERM U1219-Bordeaux Population Health, CHU de Bordeaux, Pole de santé publique, Service d’information médicale, Bordeaux, France; 11 Stichting HIV Monitoring, Amsterdam, the Netherlands, and Department of Global Health, Academic Medical Center, Amsterdam, the Netherlands; 12 Robert Koch Institute, Department for Infectious Disease Epidemiology, Berlin, Germany; 13 Hospital Clinico San Carlos, IdISSC/Universidad Complutense, Madrid, Spain; 14 Epidemiology Service, Public Health Agency of Barcelona, Barcelona and CIBER Epidemiología y Salud Pública (CIBERESP), Madrid, Spain; 15 Istituto Nazionale Malattie Infettive L. Spallanzani, IRCCS, Roma, Italy; 16 Infectious Diseases Unit, Department of Health Sciences, ASST Santi Paolo e Carlo, University Hospital, Milan, Italy; 17 Clinic of Infectious diseases, University of Modena and Reggio Emilia, Modena, Italy; 18 Infectious Diseases Service, Parc Taulí Hospital Universitario and Universidad Autónoma de Barcelona, Barcelona, Spain; 19 San Raffaele Vita Salute University, Milano, Italy; 20 Infectious Diseases Service, Lausanne University Hospital, University of Lausanne, Lausanne, Switzerland; 21 INSERM, UMR_S 1136, Institut Pierre Louis d'Epidémiologie et de Santé Publique, and UPMC Université Paris 06, and Université Paris Descartes, Hôpital Cochin Hôtel-Dieu Paris, Paris, France; 22 Service de médecine interne et maladies infectieuses, CHU de Bordeaux, Université de Bordeaux, Inserm U1219, Bordeaux, France; 23 Department of Infectious Diseases, St Pierre University Hospital, Université Libre de Bruxelles, Brussels, Belgium; 24 Université Paris Sud, UMR 1184, Le Kremlin-Bicêtre, and CEA, DSV/iMETI, IDMIT, Fontenay-aux-Roses, and INSERM, U1184, Immunology of Viral Infections and Autoimmune Diseases, Le Kremlin-Bicêtre, France and Assistance Publique-Hôpitaux de Paris, Hôpital Bicêtre, Service de Médecine Interne et Immunologie clinique, Le Kremlin-Bicêtre, France; University of Pittsburgh Centre for Vaccine Research, UNITED STATES

## Abstract

**Objective:**

HIV controllers (HICs) spontaneously maintain HIV viral replication at low level without antiretroviral therapy (ART), a small number of whom will eventually lose this ability to control HIV viremia. The objective was to identify factors associated with loss of virological control.

**Methods:**

HICs were identified in COHERE on the basis of ≥5 consecutive viral loads (VL) ≤500 copies/mL over ≥1 year whilst ART-naive, with the last VL ≤500 copies/mL measured ≥5 years after HIV diagnosis. Loss of virological control was defined as 2 consecutive VL >2000 copies/mL. Duration of HIV control was described using cumulative incidence method, considering loss of virological control, ART initiation and death during virological control as competing outcomes. Factors associated with loss of virological control were identified using Cox models. CD4 and CD8 dynamics were described using mixed-effect linear models.

**Results:**

We identified 1067 HICs; 86 lost virological control, 293 initiated ART, and 13 died during virological control. Six years after confirmation of HIC status, the probability of losing virological control, initiating ART and dying were 13%, 37%, and 2%. Current lower CD4/CD8 ratio and a history of transient viral rebounds were associated with an increased risk of losing virological control. CD4 declined and CD8 increased before loss of virological control, and before viral rebounds.

**Discussion:**

Expansion of CD8 and decline of CD4 during HIV control may result from repeated low-level viremia. Our findings suggest that in addition to superinfection, other mechanisms, such as low grade viral replication, can lead to loss of virological control in HICs.

## Introduction

Natural history of HIV-1 infection is characterized by a gradual loss of CD4 cells, a persistent elevation of CD8 cells, together with an increase of HIV RNA viral load [1–3]. However, some HIV-1-positive individuals spontaneously control HIV replication in the absence of antiretroviral therapy (ART): they are referred to as HIV controllers (HICs) [4,5]. Although a large proportion of HICs do not appear to experience disease progression [6], a small number of HICs will eventually progress with CD4 decline and/or loss of virological control [7–11]. Very few studies have focused on the factors associated with loss of virological control, since the event is rarer than immunological progression in HICs [10]. Association with age, mode of HIV acquisition, total cell-associated HIV DNA, duration of HIV infection, or HCV coinfection were inconsistently identified with discrepancies on the magnitude of their effect, probably because of the limited number of HICs included in most of these studies [7–10]. Whilst low or declining CD4 counts have been associated with loss of virological control in HICs [7,8], the concomitant evolution of CD8 count during HIV control has never been investigated, despite the pivotal role of effective HIV-specific CD8 response in virological control [5,12–17].

The objectives of the present study were to describe the incidence of, and identify factors associated with, loss of virological control in 1067 HICs from the large European COHERE in Eurocoord collaboration of cohorts. We also modeled the evolution of CD4 counts, CD8 counts and CD4/CD8 ratio during virological control preceding the outcomes of virological control.

## Patients and methods

### Eligible population in the COHERE dataset

The Collaboration of Observational HIV Epidemiological Research Europe (COHERE) is a collaboration of 40 HIV cohorts (circa 2015) within the EuroCoord network (www.cohere.org; www.Eurocoord.net) representing over 330,000 HIV-1-positive individuals. The HIV controllers project was endorsed by 28 cohorts and the dataset pooled in 2014 included 216,614 patients.

Eligible HIV-positive individuals from the COHERE database were those who had at least 5 HIV RNA viral load (VL) measurements, were followed for more than 5 years after HIV diagnosis, were enrolled after 01/01/1996 and less than 8 years after HIV diagnosis and were aged 15 years or more at diagnosis. Enrolment date was defined as the earliest of the date of first CD4 cell counts or first VL measurement.

### Definition of HIV controllers

HICs were eligible individuals who had at least 5 consecutive VL ≤500 copies/mL whilst ART-naive, over a period of at least 1 year, with the last VL ≤500 copies/mL being at least 5 years after HIV diagnosis. These criteria were based on the ANRS definition of HICs [18]. This definition also allowed to identify HIV controllers followed at calendar periods before assays with thresholds of 40 copies became widely available. The first of the VL ≤500 copies/mL defined the start of the observed period of HIV virological control. The 5^th^ consecutive VL ≤500 copies/mL, or the n^th^ (n>5) if the 5^th^ was within 1 year after the 1^st^ VL ≤500 copies/mL or less than 5 years after HIV diagnosis, defined the date of confirmation of HIC status [19]. Virological control ended at the first of the following events: 1) confirmed loss of virological control defined as 2 consecutive VL >2000 copies/mL, 2) ART initiation, 3) death or 4) end of follow-up while still experiencing virological control.

Individuals who experienced within a 6 month-period a spontaneous drop of VL at least 2 log_10_ copies/mL from an initial VL >5.3 log_10_ copies/mL, concomitantly with an increase of at least 100 CD4/mm^3^, were suspected of having received unreported ART and were not considered as HIC. Similarly, individuals with an AIDS-related illness before confirmation of HIC status were not considered as HICs as they were expected to have initiated ART.

### Statistical analyses

Data were expressed as median (interquartile range (IQR)) for continuous variables and % (n) for categorical variables.

Loss of virological control, ART initiation and death were considered as competing outcomes in this study. The probabilities [95% confidence interval (CI)] of loss of virological control, ART initiation or death at different times after confirmation of HIC status were estimated using cumulative incidence functions, based on the Fine and Gray model for handling competing events in a survival analysis [20].

Factors associated with loss of virological control, or with ART initiation, were identified using cause-specific Cox models, with right-censoring of follow-up in individuals having experienced one of the other competing events. Cox models were stratified by cohort and set with the origin at confirmation of HIC status. In addition to baseline covariates, time-updated categorical covariates were included in the analysis to estimate the effect of current age (≤50 and >50 years), current calendar period of follow-up (<2003, 2003–2007 and >2007), current CD4 count (≤350, 351–500 and >500 cells/mm^3^), current CD8 count (≤600, 601–1200, >1200 cells/mm^3^) and current CD4/CD8 ratio (≤0.50, 0.51–0.80, 0.81–1 and >1) on the outcome. Current CD4, CD8 and CD4/CD8 values were defined as the most recent available values during virological control in a time-updated manner. Of note, 7 cohorts did not report CD8 counts. VL >500 copies/mL during virological control were defined as viral rebounds. Any viral rebound followed by a VL ≤500 copies/mL during virological control was designated as a ‘transient’ rebound. A time-updated variable describing the history of viral rebounds took the following values: 0 before any rebound, 1 after the first transient rebound, 2 after subsequent transient rebounds, and 3 during a rebound (S1 Fig); this variable was considered as a categorical variable in the models. All variables with univariate p-value <0.1 were included in the multivariate models.

Cox models correcting for competing events using the Fine and Gray method [21] or inverse probability weighting [22,23] were also performed and produced similar results. Sensitivity analyses were conducted: i) restricting the analysis to men in order to remove women who would have initiated ART for the sole purpose of prevention of mother-to-child transmission, and ii) excluding HICs with less frequent VL monitoring during virological control (with at least one time interval between 2 consecutive VLs that was greater than 2 years).

The dynamics of CD4 count, CD8 count and the CD4/CD8 ratio during virological control were described using mixed-effect linear models in order to take into account the correlation between measurements from the same patient. Square root transformations for CD4 and CD8 counts were used to fulfill the model assumptions. The models included fixed and random effects for both the intercept and slopes (unstructured covariance). The CD4, CD8 and CD4/CD8 dynamics were described over the last five years preceding loss of virological control, ART initiation, death, or censoring. Finally, in order to investigate the association between the occurrence of viral rebounds and the evolution of immunologic markers during virological control, the dynamics of CD4 count, CD8 count and CD4/CD8 were compared before and after occurrence of the first viral rebound; in that case, the study period was restricted to the 5 years before and 2 years after the first viral rebound. For these analyses, only HICs with both CD4 and CD8 values available over the relevant periods were considered. However, we verified that the predicted CD4 dynamics in these models were similar with respect to the mean intercept and slope estimates when all HICs were considered.

All analyses were performed using Stata 14 (StataCorporation, College Station, Texas, USA), but the cumulative incidence functions were estimated using R (https://R-project.org).

### Ethical considerations

In COHERE, data storage, management and handling are protected in accordance with the European Commission Directive 95/46/EC and appropriate national regulations. Ethical requirements fall within the individual cohort’s ethics.

## Results

### Characteristics of HIV controllers

We identified 1067 HICs among the 111,073 eligible individuals (0.96%) (S2 Fig). Table 1 shows the main characteristics of HICs and non-HICs. The proportion of women was higher among HICs than non HICs (42.2% and 29.2%, respectively), and the proportion of men having sex with men (MSM) among men was lower (48.8% and 56.1%, respectively) (Table 1). The proportion of injecting drug users (IDU) was slightly higher among HICs (12.3% and 10.4%, respectively). Median age at HIV diagnosis for HICs and non-HICs was 33 and 34 years, respectively. At enrolment, CD4 counts were higher and CD8 counts lower among HICs than non-HICs (669 and 333 CD4/mm^3^, and 788 and 850 CD8/mm^3^, respectively). Median CD4/CD8 ratio was higher among HICs than non-HICs (0.84 and 0.36, respectively). Only 15,552 (14%) of the 111,073 eligible individuals had test results available to determine their HCV status in COHERE database. Among those with a known HCV status, HIV-HCV coinfection was identified in 69.7% of HICs and 59.5% of non-HICs (data not shown).

**Table 1:** Characteristics of HIV controllers and non-HIC from COHERE eligible patients.

		HICs	Non-HICs
		N = 1067	N = 110,006
		% (n)	% (n)
**Women**		42.2 (450)	29.2 (32,099)
**Mode of HIV acquisition**			
	IDU	12.3 (131)	10.4 (11,436)
	MSM	28.2 (301)	39.7 (43,671)
	Other sexual contact	48.9 (522)	42.4 (46,686)
	Other or unknown	10.6 (113)	7.5 (8,213)
**Age at HIV diagnosis (years)**		33 (28–40)	34 (28–41)
**Duration of follow-up (years)**		8.5 (6.3–11.9)	8.9 (6.4–12.2)
**CD4 cell count at enrolment (cells/mm**^**3**^**)**^**a**^		669 (490–887)	333 (163–520)
**CD8 cell count at enrolment (cells/mm**^**3**^**)**^**b**^		788 (587–1092)	850 (584–1210)
**CD4/CD8 ratio at enrolment (cells/mm**^**3**^**)**^**c**^		0.84 (0.58–1.18)	0.36 (0.19–0.60)

Data are median (IQR) or % (n); HICs, HIV controllers; IDU, injecting drug users; MSM, men having sex with men.

^a^ Closest CD4 measurement available in the following 6 months, 27 and 2997 missing values among HIC and non-HIC

^b^ Closest CD8 measurement available in the following 6 months, 388 and 43,763 missing values among HIC and non-HIC

^c^ Closest CD4/CD8 values available in the following 6 months, 389 and 43,763 missing values among HIC and non-HIC

Among the 1067 HICs, 86 experienced loss of virological control, 293 initiated ART and 13 died during virological control (3 from non-AIDS defining infections, 2 cardiovascular diseases, 1 chronic hepatitis C, 1 carcinoma, 1 mental disorder related to drug abuse and 5 unknown/unreported causes of death). The remaining 675 HICs maintained virological control up to the end of follow-up and their follow-up was therefore right-censored.

**Fig 1:**
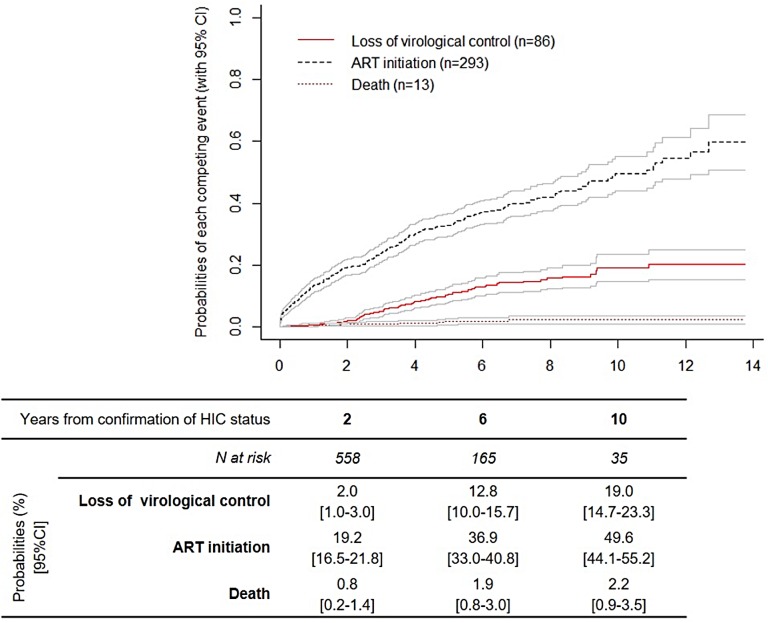
Cumulative incidence functions for loss of virological control, ART initiation and death since confirmation of HIV controller status.

The probabilities of loss of virological control 2, 6 and 10 years after confirmation of HIC status were 2.0%, 12.8% and 19.0%, respectively (Fig 1). The corresponding probabilities for ART initiation and death during virological control are illustrated in Fig 1. Altogether, the probabilities of maintaining HIC status 2, 6 and 10 years after confirmation of HIC status were 78.0% [75.1–80.7], 48.4% [44.1–52.6] and 29.2% [23.6–34.9], respectively.

During virological control, 310 HICs (29.1%) experienced viral rebounds, including 167 (15.7%) with transient rebounds (Table 2). The median delay from the confirmation of HIC status to the first rebound was 1.2 years in those who experienced rebounds. The median (IQR) maximum level reached during viral rebound was 1140 (710–1980) copies/ml.

**Table 2:** Characteristics according to the outcome of virological control in 1067 HIV controllers from COHERE Collaboration.

			Outcome of HIV control			
Characteristics		Total HICs	Loss of virological control	ART initiation	Death	Censored
		N = 1067	N = 86	N = 293	N = 13	N = 675
**History of viral rebounds**						
	No rebounds	70.9 (757)	24.4 (21)	64.5 (189)	69.2 (9)	79.7 (538)
	Only transient rebound(s)	9.2 (98)	10.5 (9)	13.3 (39)	7.7 (1)	7.3 (49)
	Only 1 non-completed rebound	13.4 (143)	46.5 (40)	14.0 (41)	7.7 (1)	9.0 (61)
	Both type of rebounds	6.5 (69)	18.6 (16)	8.2 (24)	15.4 (2)	4.0 (27)
**Years from confirmation of HIC status to 1**^**st**^ **rebound** ^**a**^		1.2 (0.6–2.5)	1.1 (0.6–1.8)	1.2 (0.6–2.7)	0.6 (0.6–0.9)	1.4 (0.7–3.0)
**CD4 count at outcome (cells/mm**^**3**^**)** ^**b**^		614 (437–850)	515 (428–656)	412 (305–600)	527 (453–867)	711 (540–950)
**CD8 count at outcome (cells/mm**^**3**^**)** ^**b**^		835 (594–1210)	1006 (770–1437)	780 (545–1110)	714 (409–1420)	837 (588–1224)
**CD4/CD8 ratio at outcome** ^**b**^		0.73 (0.47–1.14)	0.49 (0.36–0.66)	0.54 (0.36–0.93)	0.77 (0.60–1.55)	0.86 (0.58–1.30)

Data are median (IQR) or % (n); HICs, HIV controllers.

^a^ Among 310 HICs who experienced rebounds

^b^ Closest CD4, CD8 or CD4/CD8 available value within the 3 month-period preceding end of virological control or censoring

During virological control, 4 HICs had no CD4 measurements and 261 HICs had no CD8 measurements. In HICs with measurements, the median (IQR) number of VL, CD4 and CD8 measurements during virological control were 11 (8–16), 12 (8–17) and 11 (7–16), respectively; the median time interval between each pair of consecutive measurements for VL, CD4 and CD8 was 5.0 (3.4–6.9), 4.7 (3.3–6.5) and 4.6 (3.2–6.5) months, respectively.

Median CD4 counts at the end of the period of virological control were lower in those who lost virological control, initiated ART or died, compared to those still in virological control (515, 412, 527, and 711 CD4/mm^3^, respectively) (Table 2). Median CD8 counts were higher in those who lost virological control compared to those who initiated ART, died or remained in virological control (1006, 780, 714, and 837 CD8/mm^3^, respectively).

### Factors associated with loss of virological control

In univariate Cox analysis, men were at a higher risk of losing virological control than women (Table 3). Our large sample of HIC allowed us to attempt to unravel the role of gender from the role of the mode of HIV acquisition. Compared to non-IDU women, IDU men or IDU women did not appear to be at greater risk of losing virological control, while non-IDU men (either MSM or MSW) were at higher risk of losing virological control. Of note, median (IQR) time between VL measurements during virological control was 4.9 (3.4–6.7) months in non-IDU men and 5.1 (3.5–7.1) months in non-IDU women (p = 0.01). This could have introduced a bias as non-IDU men were monitored more frequently than non-IDU women, which in turn could have explained the greater odds of loss of control in non-IDU men. However, in a sensitivity analysis excluding HICs with less frequent VL monitoring during virological control (at least one time interval between 2 consecutive VLs> 2 years), similar results were obtained, i.e. non-IDU men were at a higher risk of losing virological control than non-IDU women. When compared to those with a CD4 >500 cells/mm^3^, individuals with a current CD4 count of 351–500 cells/mm^3^ had an increased risk of losing virological control, while those with a current CD4 count ≤350 cells/mm^3^ did not. Individuals with higher current CD8 counts had an increased risk of losing virological control. Individuals with a current CD4/CD8 ratio between 0.51 and 0.80 had an increased risk of losing virological control when compared to those with a ratio >1; the risk was even higher among those with a ratio ≤0.5. The risk of loss of virological control was increased after the 1^st^ transient viral rebound, compared with no rebound, and was even higher after subsequent transient rebounds. Age at diagnosis, current age and current period of follow-up were not associated with increased risk of losing virological control.

**Table 3:** Factors associated with loss of virological control in 1067 HIV controllers from the COHERE Collaboration.

		1067 HICs		Univariate analysis *		Multivariate analysis *	
		person-years (3173)	Virological loss (n = 86)	Crude HR [95% CI]	p	Adjusted HR [95% CI]	P
**Gender**					0.013		
	Women	1381	27	1			
	Men	1792	59	1.81 [1.12–2.94]			
**Mode of HIV acquisition by gender**					0.031		0.11
	IDU women	118	3	1.13 [0.32–4.04]		1.53 [0.31–7.61]	
	IDU men	331	5	0.68 [0.25–1.87]		0.51 [0.15–1.73]	
	MSM	933	36	2.11 [1.20–3.71]		2.24 [1.15–4.34]	
	MSW	383	14	2.16 [1.08–4.34]		1.28 [0.51–3.23]	
	Non-IDU women	1161	24	1		1	
	Other or unknown	247	4	0.95 [0.32–2.85]		1.41 [0.39–5.04]	
**Current age ‡**					0.59		
	≤50 years	2515	69	1			
	>50 years	658	17	0.86 [0.48–1.52]			
**Current period of follow-up ‡**					0.42		
	<2003	384	11	1			
	2003–2007	1314	36	0.61 [0.29–1.29]			
	>2007	1475	39	0.61 [0.28–1.29]			
**Current CD4 level (cells/mm^3^)‡**					0.006		0.008
	>500	2383	50	1		1	
	351–500	542	27	2.36 [1.42–3.90]		2.57 [1.41–4.67]	
	≤350	240	8	1.48 [0.68–3.21]		1.32 [0.53–3.30]	
**Current CD8 level (cells/mm**^**3**^**) ‡**					0.016		0.26
	≤600	565	6	1		1	
	601–1200	1247	36	2.46 [1.02–5.92]		1.33 [0.53–3.30]	
	>1200	574	24	3.38 [1.35–8.44]		1.98 [0.76–5.15]	
**Current CD4/CD8 ratio level ‡**					<0.0001		
	>1	873	10	1			
	0.81–1	307	4	1.02 [0.31–3.34]			
	0.51–0.80	680	26	3.26 [1.55–6.87]			
	≤0.50	523	26	4.48 [2.10–9.56]			
**History of viral rebounds ‡**					<0.0001		<0.0001
	Before any rebound	2533	21	1		1	
	After the 1^st^ transient rebound	193	5	2.84 [1.04–7.79]		1.90 [0.53–6.82]	
	After subsequent transient rebounds	53	4	7.99 [2.32–27.46]		9.24 [2.42–35.36]	
	During a rebound	394	56	12.95 [7.49–22.37]		12.15 [6.38–23.13]	

HICs, HIV controllers; IDU, injecting drug users; MSM, men having sex with men; MSW, men having sex with women.

* Stratified on cohort

‡ Time-updated covariates

In multivariate analysis, transient viral rebounds, a current CD4 count of 351–500 cells/mm^3^ and MSM mode of acquisition remained independently associated with an increased risk of losing virological control (Table 3). Higher CD8 counts were no longer statistically significant after adjustment for viral rebounds. In sensitivity analysis restricted to men, transient viral rebounds and a current CD4 count between 351 and 500 cells/mm^3^ were associated with a higher risk of losing virological control in multivariate analysis.

### Factors associated with ART initiation

Since ART initiation was a major competing event (n = 293), factors associated with this outcome were investigated. In univariate Cox analysis, decreasing current CD4 levels were associated with increasing chance of ART initiation, as expected. Lower current CD4/CD8 ratio and more recent calendar period were also associated with a higher chance of ART initiation (S1 Table). Individuals with transient viral rebounds were more likely to initiate ART, although the HRs were lower than those observed for loss of virological control. Current CD8 level was not associated with an increased uptake of ART.

### CD4 and CD8 dynamics during virological control

For 806 HICs who had both CD4 and CD8 measurements during the whole period of virological control, the mean CD4 and CD8 levels at the observed start of virological control, estimated with a linear mixed effect model, were 26.51 √CD4/mm^3^ (i.e. 703 CD4/mm^3^) and 29.46 √CD8/mm^3^ (i.e. 868 CD8/mm^3^) respectively. Overall, during virological control, CD4 counts decreased significantly by a mean of -0.16 √cells/mm^3^/year (p<0.001) whereas CD8 counts tended to increase (+0.06 √cells/mm^3^/year; p = 0.07). The estimated mean CD4/CD8 ratio was 0.94 at the beginning of the period of virological control and decreased significantly by -0.01 per year (p<0.001).

### CD4 and CD8 dynamics according to outcome of virological control

**Fig 2:**
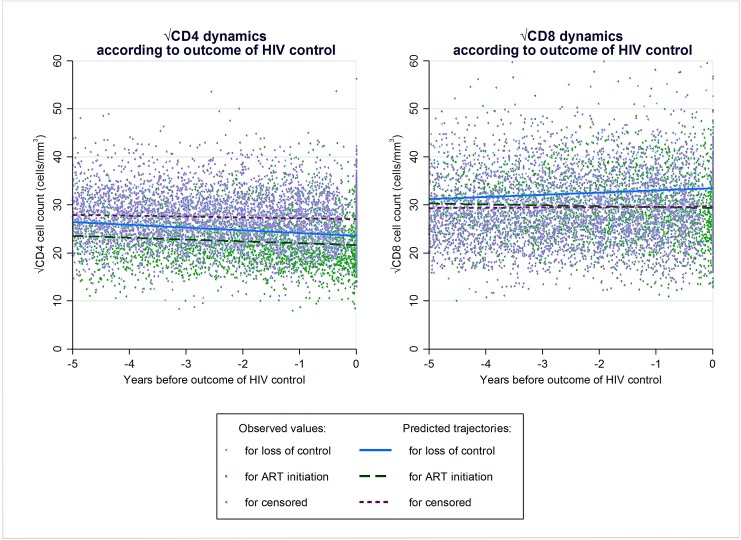
Immunologic evolution during the last 5 years of HIV control according to the event ending virological control (in 794 HIV controllers with both CD4 and CD8 measurements during this period).

The progression of CD4, CD8 and CD4/CD8 ratio over the 5 years preceding the end of virological control, or end of follow-up for those who maintained virological control, were described (Fig 2) (S2 Table) in 794 HICs with both CD4 and CD8 measurements available. CD4, CD8 and CD4/CD8 slopes were not significantly different from zero in those who died during virological control but the sample was small (n = 9). Mean CD4 decline was significantly greater in those who initiated ART (n = 227) or lost virological control (n = 66) than in those who maintained virological control at the end of follow-up (n = 492). At the end of the period of virological control, CD4 level was lower in those who initiated ART than in those who lost virological control, and was higher in those who maintained virological control. Mean CD8 counts increased significantly in those who experienced loss of virological control whereas it remained stable in the other groups. The CD8 level at the end of the period of virological control was much higher in those who experienced loss of virological control. Mean CD4/CD8 ratio also decreased significantly in those who experienced loss of virological control, and to a lesser extent in those who had maintained virological control at the end of follow-up. The ratio was stable in those who initiated ART.

We further investigated if the rates of change of CD4, CD8 or CD4/CD8 differed during the year preceding loss of virological control as compared to prior this one-year period. We could not identify a significant change of the slope for CD8 or for CD4/CD8 ratio. On the other hand, during the year preceding loss of virological control, the CD4 decrease was significantly steeper than during the period further before (-1.51 √cells/mm^3^/year and -0.38 √cells/mm^3^/year, respectively; p = 0.026).

### CD4 and CD8 dynamics according to occurrence of viral rebounds

For 247 HICs who experienced viral rebounds during virological control, we investigated CD4 and CD8 dynamics before and after the occurrence of the first viral rebound. We restricted the analysis to the 5 years before and 2 years after the first viral rebound. Mean CD4 counts decreased significantly prior to the first viral rebound by -0.52 √cells/mm^3^/year and continued to decrease at the same pace afterwards (comparison of 1^st^ and 2^nd^ slopes: p = 0.74). Similarly, mean CD8 counts increased significantly prior to occurrence of the first viral rebound by +0.31 √cells/mm^3^/year and continued to increase at the same pace afterwards (p = 0.56). Mean CD4/CD8 ratio decreased significantly prior to the first viral rebound by -0.037 per year and continued to decrease at the same pace afterwards (p = 0.44). We also investigated if the rates of change of CD4, CD8 or CD4/CD8 differed during the year preceding the first viral rebound as compared to prior this one-year period. We could not identify a significant change of the slope for CD8 or for CD4/CD8 ratio. Regarding the CD4 progression, the decrease was significantly steeper during the year preceding the first rebound compared to earlier (p<0.001).

## Discussion

In this study we identified 1067 HICs, representing 1% of eligible individuals. This is, to date, the largest series of HIV controllers, internationally. This allowed us to study the incidence of and risk factors for loss of virological control, which had rarely been done. Over a follow-up period to 2014, the risk of losing virological control remained relatively low over time, compared with the main competing event signifying the end of the period of virological control, i.e. ART initiation. For example, 6 years after confirmation of HIC status, the probability for HICs of maintaining virological control was 48%, with a probability of losing virological control of 13% and a much higher probability of initiating ART of 37%.

Men, either MSM or MSW, were at a higher risk of losing virological control than non-IDU women or IDU men and women in univariate analysis, even when restricting the analysis to HIC with regular VL monitoring. Some MSW in our study may have chosen not to report a sexual preference for men, but it is unlikely that any under-reporting would be large enough to explain this finding. We and other authors previously described an over-representation of IDUs and an under-representation of MSM among HICs compared to non-HICs [16,6]; another HIC study also found that MSM were at greater risk of virologic and immunologic progression than IDU [7]. A potential explanation for this increased risk could be a higher risk of sexually acquired super-infection, which has been reported to lead to loss of control among elite controllers [25–27], or active coinfections. In natural history studies, women have been reported to have lower viral load than men at the same time after infection [28,29]. We can therefore hypothesize that women maintain this advantage during spontaneous virological control.

Other factors associated with the risk of losing virological control were a current CD4 count between 350 and 500/mm^3^, current higher CD8 counts or lower CD4/CD8 ratio, and a history of transient viral rebounds during control. Of note, a CD4 level below 350 cells/mm^3^ was not associated with an increased risk of losing virological control, most likely because HICs presenting with low CD4 counts initiated ART before loss of virological control could be observed, in line with guidelines [30,31]. In multivariate analysis, a current CD4 count between 350 and 500/mm^3^ and a history of transient viral rebounds remained independently associated with the risk of losing virological control.

Modeling CD4 and CD8 counts during the 5 years preceding the end of virological control allowed us to describe with greater precision the dynamics of these cells before the outcome. A significant CD8 count increase was observed in those who subsequently lost virological control but not in the other groups; this finding has never been described to date. We observed a similar CD4 count decrease in those who lost control or initiated ART, with the latter group having lower absolute counts. Interestingly, CD4 counts also decreased in those who maintained virological control at the end of follow-up, around 9 cells/year, albeit at a slower pace than during natural history [32]. The association between CD4 decline and occurrence of blips during HIV control has been previously described [9,10,12], but here, and for the first time to our knowledge, we were able to model lymphocyte count dynamics before and after viral rebounds. We showed that CD4 counts and CD4/CD8 ratio declined several years before the first viral rebound was clinically observed. These findings suggest that super-infections are not the only mechanism leading to loss of control in HICs.

Our results are consistent with a role of chronic immune activation, inversely correlated with CD4 cell counts and positively correlated with CD8 counts. It was recently shown that high levels of T cell activation and inflammation parameters were associated with disease progression among HICs [10]. Viral rebound history and CD4 decline were suggested to be the consequence of a persistent low-level viral replication in studies investigating immunologic progression during HIV control [10,24,33,34]. Higher CD8 counts at ART initiation or an increase in CD8 count during ART were predictive factors of virological treatment failure [35,36]. Elevated CD8 counts and decreasing CD4 counts may result from persistent or repeated low-level viremia.

One limitation of our study is the lack of confirmation of ART-naïve status as no drug dosage was systematically conducted. However, to prevent considering patients on ART as HICs, we removed those who experienced suspicious concomitant and large VL decrease and CD4 increase while no ART was declared on the database. Another limitation is that we could not study the role of different HIV subtypes on control duration since data were not complete. Finally, it is possible that the history of viral rebounds is not complete in this database, given the irregular VL monitoring in some patients. However, the CD4 level may have provided information regarding the risk of virological progression in these individuals.

The evolution of recommendations regarding ART initiation may reduce the chance to conduct studies on such a large number of HICs in the future. From a public health perspective, loss of virological control as well as occurrence of viral blips in these HICs, plead for reinforced recommendation of condom use in case of sexual contact, regardless of the HIV status of the partners. From a clinical perspective, careful monitoring of untreated HICs who experience decrease of CD4 and CD4/CD8 ratio during virological control, or transient viral rebounds, is needed.

## Supporting information

S1 Fig Time-updated variable describing the history of viral rebounds during HIV control. (TIF)

S2 Fig Flow chart of selection of HICs. (TIF)

S1 Table Factors associated with ART initiation. (DOCX)

S2 Table CD4 and CD8 dynamics during the last 5 years of HIV control preceding the end of virological control of the of follow-up in 794 HICs from the COHERE Collaboration. (DOCX)

## References

[1] Huang X, Lodi S, Fox Z, Li W, Phillips A, Porter K, et al Rate of CD4 decline and HIV-RNA change following HIV seroconversion in men who have sex with men: a comparison between the Beijing PRIMO and CASCADE cohorts. J Acquir Immune Defic Syndr 2013;62:441–6. 10.1097/QAI.0b013e31827f5c9a 23221982

[2] Hubert JB, Burgard M, Dussaix E, Tamalet C, Deveau C, Le Chenadec J, et al Natural history of serum HIV-1 RNA levels in 330 patients with a known date of infection. The SEROCO Study Group. AIDS 2000;14:123–31. 10708282 10.1097/00002030-200001280-00007

[3] Cao W, Mehraj V, Kaufmann DE, Li T, Routy J-P. Elevation and persistence of CD8 T-cells in HIV infection: the Achilles heel in the ART era. J Int AIDS Soc 2016;19:20697 10.7448/IAS.19.1.20697 26945343 PMC4779330

[4] Lambotte O, Boufassa F, Madec Y, Nguyen A, Goujard C, Meyer L, et al HIV controllers: a homogeneous group of HIV-1-infected patients with spontaneous control of viral replication. Clin Infect Dis 2005;41:1053–6. 10.1086/433188 16142675

[5] Deeks SG, Walker BD. Human Immunodeficiency Virus Controllers: Mechanisms of Durable Virus Control in the Absence of Antiretroviral Therapy. Immunity 2007;27:406–16. 10.1016/j.immuni.2007.08.010 17892849

[6] Okulicz JF, Lambotte O. Epidemiology and clinical characteristics of elite controllers. Curr Opin HIV AIDS 2011;6:163–8. 10.1097/COH.0b013e328344f35e 21502920

[7] Leon A, Perez I, Ruiz-Mateos E, Benito JM, Leal M, Lopez-Galindez C, et al Rate and predictors of progression in elite and viremic HIV-1 controllers. AIDS 2016;30:1209–20. 10.1097/QAD.0000000000001050 26854807

[8] Madec Y, Boufassa F, Porter K, Meyer L. Spontaneous control of viral load and CD4 cell count progression among HIV-1 seroconverters. AIDS 2005;19:2001–7. 16260907 10.1097/01.aids.0000194134.28135.cd

[9] Madec Y, Boufassa F, Porter K, Prins M, Sabin C, d’Arminio Monforte A, et al Natural history of HIV-control since seroconversion. AIDS 2013;27:2451–60. 10.1097/01.aids.0000431945.72365.01 23912979

[10] Noel N, Lerolle N, Lécuroux C, Goujard C, Venet A, Saez-Cirion A, et al Immunologic and Virologic Progression in HIV Controllers: The Role of Viral “Blips” and Immune Activation in the ANRS CO21 CODEX Study. PLoS One 2015;10:e0131922 10.1371/journal.pone.0131922 26146823 PMC4493076

[11] Okulicz JF, Marconi VC, Landrum ML, Wegner S, Weintrob A, Ganesan A, et al Clinical outcomes of elite controllers, viremic controllers, and long-term nonprogressors in the US Department of Defense HIV natural history study. J Infect Dis 2009;200:1714–23. 10.1086/646609 19852669

[12] Hunt PW, Brenchley J, Sinclair E, McCune JM, Roland M, Page‐Shafer K, et al Relationship between T Cell Activation and CD4 + T Cell Count in HIV‐Seropositive Individuals with Undetectable Plasma HIV RNA Levels in the Absence of Therapy. J Infect Dis 2008;197:126–33. 10.1086/524143 18171295 PMC3466592

[13] Peris-Pertusa A, López M, Rallón NI, Restrepo C, Soriano V, Benito JM. Evolution of the functional profile of HIV-specific CD8+ T cells in patients with different progression of HIV infection over 4 years. J Acquir Immune Defic Syndr 2010;55:29–38. 10.1097/QAI.0b013e3181e69609 20634703

[14] Sáez-Cirión A, Lacabaratz C, Lambotte O, Versmisse P, Urrutia A, Boufassa F, et al HIV controllers exhibit potent CD8 T cell capacity to suppress HIV infection ex vivo and peculiar cytotoxic T lymphocyte activation phenotype. Proc Natl Acad Sci U S A 2007;104:6776–81. 10.1073/pnas.0611244104 17428922 PMC1851664

[15] Sáez-Cirión A, Pancino G, Sinet M, Venet A, Lambotte O. HIV controllers: how do they tame the virus? Trends Immunol 2007;28:532–40. 10.1016/j.it.2007.09.002 17981085

[16] Walker BD, Yu XG. Unravelling the mechanisms of durable control of HIV-1. Nat Rev Immunol 2013;13:487–98. 10.1038/nri3478 23797064

[17] Pereyra F, Addo MM, Kaufmann DE, Liu Y, Miura T, Rathod A, et al Genetic and immunologic heterogeneity among persons who control HIV infection in the absence of therapy. J Infect Dis 2008;197:563–71. 10.1086/526786 18275276

[18] Sáez-Cirión A, Hamimi C, Bergamaschi A, David A, Versmisse P, Mélard A, et al Restriction of HIV-1 replication in macrophages and CD4+ T cells from HIV controllers. Blood 2011;118:955–64. doi:10.1182/blood-2010-12-327106. 10.1182/blood-2010-12-327106 21642597 PMC3148172

[19] Suissa S. Immortal time bias in pharmaco-epidemiology. Am J Epidemiol 2008;167:492–9. 10.1093/aje/kwm324 18056625

[20] Fine JP, Gray RJ. A Proportional Hazards Model for the Subdistribution of a Competing Risk. J Am Stat Assoc 2012.

[21] Geskus RB. Data Analysis with Competing Risks and Intermediate States. Vol. 82 Boca Raton: CRC Press Book; 2015.

[22] Wal WM van der, Geskus RB. ipw: An R Package for Inverse Probability Weighting. J Stat Softw 2011;43:1–23.

[23] van Geloven N, Geskus RB, Mol BW, Zwinderman AH. Correcting for the dependent competing risk of treatment using inverse probability of censoring weighting and copulas in the estimation of natural conception chances. Stat Med 2014;33:4671–80. 10.1002/sim.6280 25088060

[24] Boufassa F, Saez-Cirion A, Lechenadec J, Zucman D, Avettand-Fenoel V, Venet A, et al CD4 dynamics over a 15 year-period among HIV controllers enrolled in the ANRS French observatory. PLoS One 2011;6:e18726 10.1371/journal.pone.0018726 21533035 PMC3080877

[25] Clerc O, Colombo S, Yerly S, Telenti A, Cavassini M. HIV-1 elite controllers: beware of super-infections. J Clin Virol 2010;47:376–8. 10.1016/j.jcv.2010.01.013 20153976

[26] Rachinger A, Navis M, van Assen S, Groeneveld PHP, Schuitemaker H. Recovery of viremic control after superinfection with pathogenic HIV type 1 in a long-term elite controller of HIV type 1 infection. Clin Infect Dis 2008;47:e86–9. 10.1086/592978 18947331

[27] Goujard C, Chaix M, Lambotte O, Deveau C, Sinet M, Guergnon J, et al Spontaneous Control of Viral Replication during Primary HIV Infection: When Is “HIV Controller” Status Established? Clin Infect Dis 2009;49:982–6. 10.1086/605504 19681706

[28] Prins M, Meyer L, Hessol NA. Sex and the course of HIV infection in the pre- and highly active antiretroviral therapy eras. AIDS 2005;19:357–70. 15750389 10.1097/01.aids.0000161765.75663.27

[29] Cabrera-Muñoz E, Hernández-Hernández OT, Camacho-Arroyo I. Role of estradiol and progesterone in HIV susceptibility and disease progression. Mini Rev Med Chem 2012;12:1049–54. 22827217 10.2174/138955712802762185

[30] European AIDS Clinical Society. Guidelines 2015;Version 8. http://www.eacsociety.org/guidelines/eacs-guidelines/eacs-guidelines.html (accessed April 23, 2016).

[31] WHO. Consolidated Guidelines on the Use of Antiretroviral Drugs for Treating and Preventing HIV Infection: Recommendations for a Public Health Approach—PubMed—NCBI 2013. http://www.ncbi.nlm.nih.gov/pubmed/24716260 (accessed April 23, 2016).

[32] Lodi S, Phillips A, Touloumi G, Pantazis N, Bucher HC, Babiker A, et al CD4 decline in seroconverter and seroprevalent individuals in the precombination of antiretroviral therapy era. AIDS 2010;24:2697–704. 10.1097/QAD.0b013e32833ef6c4 20885283

[33] Pereyra F, Palmer S, Miura T, Block BL, Wiegand A, Rothchild AC, et al Persistent low-level viremia in HIV-1 elite controllers and relationship to immunologic parameters. J Infect Dis 2009;200:984–90. 10.1086/605446 19656066 PMC3725728

[34] Noel N, Boufassa F, Lécuroux C, Saez-Cirion A, Bourgeois C, Dunyach-Remy C, et al Elevated IP10 levels are associated with immune activation and low CD4^+^ T-cell counts in HIV controller patients. AIDS 2014;28:467–76. 10.1097/QAD.0000000000000174 24378753

[35] Krantz EM, Hullsiek KH, Okulicz JF, Weintrob AC, Agan BK, Crum-Cianflone NF, et al Elevated CD8 counts during HAART are associated with HIV virologic treatment failure. J Acquir Immune Defic Syndr 2011;57:396–403. 10.1097/QAI.0b013e318221c62a 21602694 PMC3173352

[36] Lu W, Mehraj V, Vyboh K, Cao W, Li T, Routy J-P. CD4:CD8 ratio as a frontier marker for clinical outcome, immune dysfunction and viral reservoir size in virologically suppressed HIV-positive patients. J Int AIDS Soc 2015;18:20052 10.7448/IAS.18.1.20052 26130226 PMC4486418

