# Supplementary figures and images for: Impact of CD4 and CD8 dynamics and viral rebounds on loss of virological control in HIV controllers
Source: PLoS One. 2017 Apr 5;12(4):e0173893. doi: 10.1371/journal.pone.0173893 (PMC5381858; doi:10.1371/journal.pone.0173893)

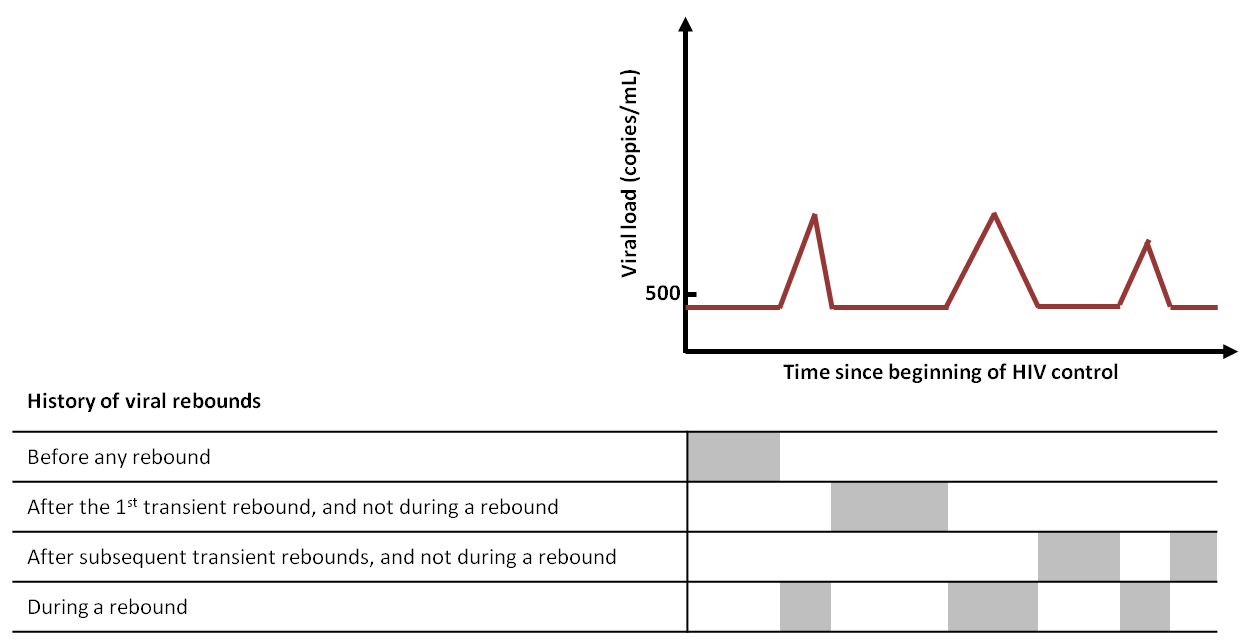

Supplement: S1 Fig — (TIF) [file pone.0173893.s001.tif]

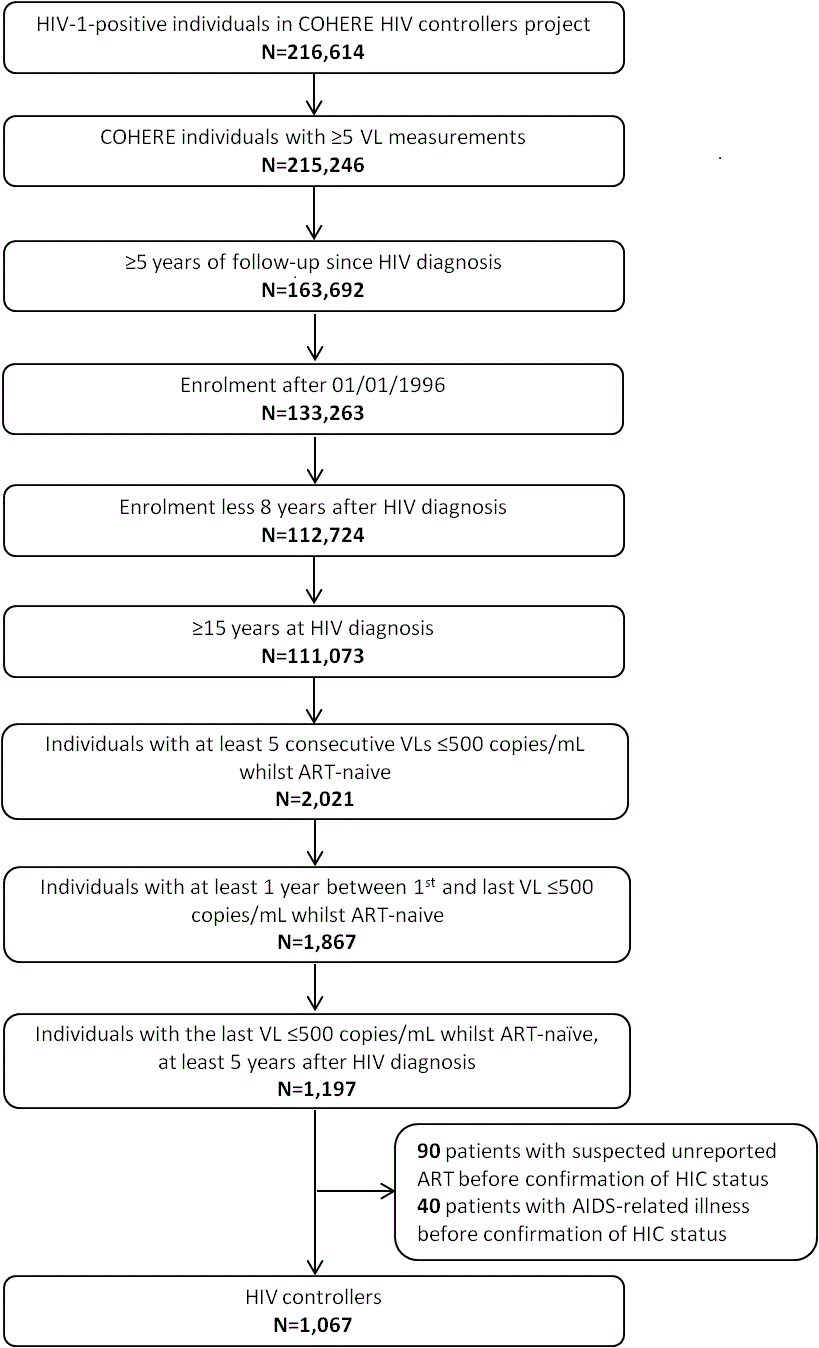

Supplement: S2 Fig — (TIF) [file pone.0173893.s002.tif]
