# Supplementary material for: Impact of CD4 and CD8 dynamics and viral rebounds on loss of virological control in HIV controllers
Source: PLoS One. 2017 Apr 5;12(4):e0173893. doi: 10.1371/journal.pone.0173893 (PMC5381858; doi:10.1371/journal.pone.0173893)
Supplement: S1 Table — (DOCX) [file pone.0173893.s003.docx]

**S1 Table: Factors associated with ART initiation**

|  | | **1067 HICs** |  | **Univariate analysis** * | | **Multivariate analysis** * | |
| --- | --- | --- | --- | --- | --- | --- | --- |
|  | | **Person-years (3173)** | **ART initiation (n=293)** | **Crude HR [95% CI]** | **p** | **Adjusted HR [95% CI]** | **p** |
| **Gender** | |  |  |  | 0.53 |  |  |
|  | Women | 1381 | 125 | 1 |  |  |  |
|  | Men | 1792 | 168 | 1.08 [0.85-1.37] |  |  |  |
| **Mode of HIV acquisition by gender** | |  |  |  | 0.55 |  |  |
|  | IDU women | 118 | 13 | 1.28 [0.69-2.38] |  |  |  |
|  | IDU men | 321 | 30 | 1.12 [0.72-1.73] |  |  |  |
|  | MSM | 933 | 75 | 1.00 [0.73-1.37] |  |  |  |
|  | MSW | 383 | 37 | 1.05 [0.71-1.55] |  |  |  |
|  | Non-IDU women | 1161 | 102 | 1 |  |  |  |
|  | Other or unknown | 247 | 36 | 1.45 [0.98-2.16] |  |  |  |
| **Current age ‡** | |  |  |  | 0.28 |  |  |
|  | ≤50 years | 2515 | 236 | 1 |  |  |  |
|  | >50 years | 658 | 57 | 0.85 [0.62-1.15] |  |  |  |
| **Current period of follow-up ‡** | |  |  |  | <0.0001 |  | <0.0001 |
|  | <2003 | 384 | 11 | 1 |  | 1 |  |
|  | 2003-2007 | 1314 | 105 | 3.03 [1.57-5.85] |  | 2.44 [1.25-4.74] |  |
|  | >2007 | 1475 | 177 | 4.58 [2.39-8.77] |  | 3.89 [2.02-7.49] |  |
| **Current CD4 level (cells/mm^3^) ‡** | |  |  |  | <0.0001 |  | <0.0001 |
|  | >500 | 2383 | 118 | 1 |  | 1 |  |
|  | 351-500 | 542 | 78 | 2.73 [2.01-3.70] |  | 2.43 [1.78-3.30] |  |
|  | ≤350 | 240 | 97 | 7.98 [5.97-10.67] |  | 7.55 [5.64-10.11] |  |
| **Current CD8 level (cells/mm^3^) ‡** | |  |  |  | 0.29 |  |  |
|  | ≤600 | 565 | 68 | 1 |  |  |  |
|  | 601-1200 | 1247 | 114 | 0.79 [0.58-1.07] |  |  |  |
|  | >1200 | 574 | 50 | 0.79 [0.54-1.16] |  |  |  |
| **Current CD4/CD8 ratio level ‡** | |  |  |  | <0.0001 |  |  |
|  | >1 | 873 | 45 | 1 |  |  |  |
|  | 0.81-1 | 307 | 25 | 1.44 [0.88-2.37] |  |  |  |
|  | 0.51-0.80 | 680 | 66 | 1.77 [1.20-2.61] |  |  |  |
|  | ≤0.50 | 523 | 96 | 3.67 [2.53-5.32] |  |  |  |
| **History of viral rebounds ‡** | |  |  |  | <0.0001 |  | <0.0001 |
|  | Before any rebound | 2533 | 207 | 1 |  | 1 |  |
|  | After the 1^st^ transient rebound | 193 | 23 | 2.14 [1.32-3.48] |  | 2.01 [1.21-3.33] |  |
|  | After subsequent transient rebounds | 53 | 7 | 1.49 [0.60-3.70] |  | 1.46 [0.59-3.65] |  |
|  | During a rebound | 394 | 56 | 2.53 [1.81-3.52] |  | 2.20 [1.55-3.12] |  |

HICs, HIV controllers; IDU, injecting drug users; MSM, men having sex with men; MSW, men having sex with women.

* Stratified on cohort

‡ Time-updated covariates
