# Supplementary material for: Impact of CD4 and CD8 dynamics and viral rebounds on loss of virological control in HIV controllers
Source: PLoS One. 2017 Apr 5;12(4):e0173893. doi: 10.1371/journal.pone.0173893 (PMC5381858; doi:10.1371/journal.pone.0173893)
Supplement: S2 Table — (DOCX) [file pone.0173893.s004.docx]

**S2 Table: CD4 and CD8 dynamics during the last 5 years of HIV control preceding the end of virological control of the of follow-up in 794 HICs from the COHERE Collaboration**

| **parameters** | **Outcome (n)** | **Estimates [95% CI]** | | | | | |
| --- | --- | --- | --- | --- | --- | --- | --- |
|  |  | **√CD4 cells/mm^3^ [95% CI]** (7428 values) | **p*** | **√CD8 cells/mm^3^ [95% CI]** (6872 values) | **p*** | **CD4/CD8 ratio [95% CI]** (6871 values) | **p*** |
| **Intercept at outcome** | Loss of control (66) | 23.58 [22.29 ; 24.87] ^a^ |  | 33.46 [31.67 ; 35.25] ^c^ |  | 0.57 [0.44 ; 0.70] ^e^ |  |
|  | ART initiation (227) | 21.66 [20.96 ; 22.35] ^b^ |  | 29.35 [28.40 ; 30.31] ^d^ |  | 0.69 [0.62 ; 0.76] ^f^ |  |
|  | Death (9) | 22.32 [18.82 ; 25.82] |  | 25.29 [20.42 ; 30.16] |  | 0.81 [0.46 ; 1.17] |  |
|  | Censored (492) | 27.03 [26.56 ; 27.51] |  | 29.63 [28.97 ; 30.28] |  | 1.01 [0.96 ; 1.05] |  |
| **Slope (per year)** | Loss of control (66) | -0.57 [-0.82 ; -0.31] ^a^ | <0.001 | 0.46 [0.10 ; 0.81] ^c^ | 0.01 | -0.042 [-0.061 ; -0.022] ^e^ | <0.001 |
|  | ART initiation (227) | -0.38 [-0.53 ; -0.23] ^b^ | <0.001 | -0.19 [-0.39 ; 0.01] ^d^ | 0.07 | -0.005 [-0.016 ; 0.006] ^f^ | 0.40 |
|  | Death (9) | -0.52 [-1.20 ; 0.16] | 0.13 | -0.41 [-1.32 ; 0.49] | 0.37 | -0.023 [-0.073 ; 0.026] | 0.35 |
|  | Censored (492) | -0.18 [-0.27 ; -0.09] | <0.001 | 0.06 [-0.06 ; 0.19] | 0.34 | -0.008 [-0.015 ; -0.001] | 0.03 |

* p value for test of slope equal to 0

^a^ difference with intercept (p<0.001) and slope (p=0.005) in censored

^b^ difference with intercept (p<0.001) and slope (p=0.025) in censored; difference with intercept (p=0.010) and slope (p=0.21) in loss of control

^c^ difference with intercept (p<0.001) and slope (p=0.039) in censored

^d^ difference with intercept (p=0.64) and slope (p=0.040) in censored; difference with intercept (p<0.001) and slope (p=0.002) in loss of control

^e^ difference with intercept (p<0.001) and slope (p=0.001) in censored

^f^ difference with intercept (p<0.001) and slope (p=0.647) in censored; difference with intercept (p=0.129) and slope (p=0.001) in loss of control
